# Heparin-Binding-Hemagglutinin-Induced IFN-γ Release as a Diagnostic Tool for Latent Tuberculosis

**DOI:** 10.1371/journal.pone.0000926

**Published:** 2007-10-03

**Authors:** Jean-Michel Hougardy, Kinda Schepers, Sammy Place, Annie Drowart, Véronique Lechevin, Virginie Verscheure, Anne-Sophie Debrie, T. Mark Doherty, Jean-Paul Van Vooren, Camille Locht, Françoise Mascart

**Affiliations:** 1 Laboratory of Vaccinology and Mucosal Immunity, Hôpital Erasme, Université Libre de Bruxelles (ULB), Brussels, Belgium; 2 Department of Pneumology, Hôpital Brugmann, Université Libre de Bruxelles (ULB), Brussels, Belgium; 3 Department of Pneumology, Hôpital Erasme, Université Libre de Bruxelles (ULB), Brussels, Belgium; 4 Department of Immunodeficiency, Hôpital Erasme, Université Libre de Bruxelles (ULB), Brussels, Belgium; 5 Department of Occupational Medicine, Hôpital Erasme, Université Libre de Bruxelles (ULB), Brussels, Belgium; 6 INSERM, U629, Lille, France; 7 Institut Pasteur de Lille, Lille, France; 8 Department of Tuberculosis Immunology, Statens Serum Institute, Copenhagen, Denmark; 9 Immunobiology Clinic, Hôpital Erasme, Brussels, Belgium; Medical Research Council Laboratories, The Gambia

## Abstract

**Background:**

The detection of latent tuberculosis infection (LTBI) is a major component of tuberculosis (TB) control strategies. In addition to the tuberculosis skin test (TST), novel blood tests, based on *in vitro* release of IFN-γ in response to *Mycobacterium tuberculosis*-specific antigens ESAT-6 and CFP-10 (IGRAs), are used for TB diagnosis. However, neither IGRAs nor the TST can separate acute TB from LTBI, and there is concern that responses in IGRAs may decline with time after infection. We have therefore evaluated the potential of the novel antigen heparin-binding hemagglutinin (HBHA) for *in vitro* detection of LTBI.

**Methodology and Principal Findings:**

HBHA was compared to purified protein derivative (PPD) and ESAT-6 in IGRAs on lymphocytes drawn from 205 individuals living in Belgium, a country with low TB prevalence, where BCG vaccination is not routinely used. Among these subjects, 89 had active TB, 65 had LTBI, based on well-standardized TST reactions and 51 were negative controls. HBHA was significantly more sensitive than ESAT-6 and more specific than PPD for the detection of LTBI. PPD-based tests yielded 90.00% sensitivity and 70.00% specificity for the detection of LTBI, whereas the sensitivity and specificity for the ESAT-6-based tests were 40.74% and 90.91%, and those for the HBHA-based tests were 92.06% and 93.88%, respectively. The QuantiFERON-TB Gold In-Tube (QFT-IT) test applied on 20 LTBI subjects yielded 50% sensitivity. The HBHA IGRA was not influenced by prior BCG vaccination, and, in contrast to the QFT-IT test, remote (>2 years) infections were detected as well as recent (<2 years) infections by the HBHA-specific test.

**Conclusions:**

The use of ESAT-6- and CFP-10-based IGRAs may underestimate the incidence of LTBI, whereas the use of HBHA may combine the operational advantages of IGRAs with high sensitivity and specificity for latent infection.

## Introduction

Tuberculosis (TB) remains a major health problem even in the 21^st^ century, with 1.6 million deaths globally recorded in 2005 by the World Health Organization, and more than 8.8 million new infections [1]. As a result, approximately 2 billion individuals are estimated to be latently infected with the causative agent, *Mycobacterium tuberculosis* [2]. Most infected subjects will not develop symptoms of the disease during their lifetime but maintain a latent TB infection (LTBI). Although the risk of progression to active disease is highest within the first two years of infection, LTBI subjects remain at life-long risk of subsequently developing the disease if not treated [3], [4]. This risk is particularly high for persons with immunosuppressive conditions, such as HIV infection, chronic renal failure, or if placed on infliximab therapy [5]–[7]. LTBI subjects thus represent an important reservoir for future TB cases, and their identification and preventive treatment or surveillance are key elements in TB control [3]–[8]. By using this strategy, eradication of TB in low-prevalence countries has been judged by some to be a realistic goal, provided that LTBI can be effectively diagnosed at a population-wide level [8].

In the absence of a gold standard, the most widely used diagnostic test for LTBI remains the tuberculin skin test (TST), based on the delayed-type hypersensitivity reaction that develops in *M. tuberculosis*-infected individuals upon intradermal injection of purified protein derivative (PPD). However, this test suffers from many limitations, including false-negative results, especially in some high-risk groups, and false-positive results in BCG-vaccinated individuals or in subjects exposed to non-tuberculous mycobacteria (NTM) [9].

More recently, two tests, based on the *in vitro* release of IFN-γ by circulating lymphocytes (hence, interferon gamma release assays or IGRAs) upon stimulation with the *M. tuberculosis*-specific antigens early secreted antigenic target, 6 kilodaltons (ESAT-6) and culture filtrate protein 10 kda (CFP-10) (the T-SPOT.*TB* test-Oxford Immunotec, Oxford, United Kingdom and the QuantiFERON-TB Gold test-QFT-G, Cellestis, Victoria, Australia), have been introduced for the diagnosis of *M. tuberculosis* infection [10], [11]. These IGRAs have been the subject of numerous studies, mostly performed on close contacts of TB index cases or on individuals with suspected TB [11]–[16], where they have been shown to be sensitive and highly specific for the detection of a recent infection. In contrast, only a few cross-sectional studies have compared the performance of IGRAs to the TST for the diagnosis of LTBI resulting from a remote infection [17], [18]. These studies suggest that IGRAs underestimate the true prevalence of LTBI, especially when resulting from a remote infection, inferring that IGRAs are most suitable for detecting recent *M. tuberculosis* infections.

In this study, we assessed the diagnostic potential of a novel mycobacterial antigen, the heparin-binding hemagglutinin (HBHA) [19], previously shown to stimulate high levels of IFN-γ secretion by the peripheral blood lymphocytes of LTBI subjects [20], [21]. A HBHA-based IGRA was compared to those based on PPD or ESAT-6, as well as to the QFT-IT test, on TST-defined, recently infected healthy subjects and on healthy subjects with a remote infection (>2 years), in comparison to non-exposed subjects and to patients with active non-treated TB.

## Methods

### Participants

235 participants were enrolled, comprising 51 negative controls (CTRL), 82 subjects with suspected LTBI and 102 patients with suspected active TB. All participants were sero-negative for HIV, and none had neoplasia, malign hemopathies or had undergone immunosuppressive treatment or solid organ transplantation, nor were any of the women pregnant during the study period. CTRL were defined on the basis of a negative TST and the absence of a history of TB or of known contact with TB patients. LTBI subjects were defined by documented TST conversion (increase in induration size of≥10 mm compared to the previous year) at the Occupational Medicine Department (n = 31) or by a diameter of induration of the TST≥10 mm for household contacts of active TB case patients (n = 21) or by the clinical data and/or TST result at the time of employment as a health care worker, as assessed by the Occupational Medicine Department (n = 13). Among the 82 subjects initially enrolled, results from 16 of them were rejected because the retrospective analysis of the clinical data indicated that these subjects did not fulfill the strict criteria for the LTBI diagnosis, and one was rejected for a technical problem in the IGRA. TST consisted of an intradermal injection of 2 tuberculin units (TU) of PPD (RT23, Statens Serum Institute, Copenhagen, Denmark), and the results of the TST were read 48 to 72 hours later. Blood samples were collected within 6 months of the TST for 34 subjects considered as recent TST converters (21 TST^+^ household contacts and 13 health care workers with a TST conversion). For the other 31 LTBI subjects included in the study, blood samples were collected at least 2 years after the TST (mean time between TST and IGRA: 14.23 years, SEM: 9.68 years). For 17 among them, 2 to 6 sequential blood tests were performed (see Table 1).

**Table 1:** Longitudinal follow-up of LTBI with the HBHA-IGRA test

LTBI subject n°	Year of diagnosis	Year of HBHA IGRA test					
		2000	2001	2002	2003	2006	2007
1	1979	+	+	+		+	+
2	1979					+	+
3	1989					+	+
4	1992	+	+	+	+	+	+
5	1993			+		+	+
6	1994					+	+
7	2000	+				+	
8	2001		+			+	
9	1986		+	+	+		
10	1985		+	+			
11	1972		+	+			
12	1978					+	+
13	1995		+	+		+	
14	2000		+		+	+	
15	2000			+			+
16	1990					+	+
17	1985			+		+	

Active TB cases were all confirmed on the basis of at least one of the criteria defined by the American Thoracic Society [22]: a positive smear and/or a positive culture, the presence of granuloma in a biopsy, a positive polymerase chain reaction or a clinical suspicion with favorable clinical response upon treatment. Of the 102 suspected TB patients initially enrolled, 13 were rejected for absence of formal proof of active TB as defined by these criteria. Among the remaining 89 TB patients, 58 presented with pulmonary TB, and 31 with extra-pulmonary TB. Of these latter, 13 had pleural effusions, whereas the others presented with adenopathies (n = 5), urinary and/or abdominal TB (n = 7), pericarditis (n = 1), uveitis and pulmonary TB (n = 1), or disseminated miliary TB (n = 4). Blood samples were taken before or during the first week of chemotherapy. All the study participants were living in Belgium at the time of enrolment. Blood samples were collected from 1999 through to 2007. Demographic data are given in Table 2. The study was approved by the Ethical Committee of the Université Libre de Bruxelles and by the Ethical Committees from three different hospitals where the patients were recruited. Informed consent was given by all participants.

**Table 2:** Characteristics of the included subjects

	CTRL	LTBI	TB
N	51	65	89
% women	72.5	61.5	20.7
Median age (yrs)	36.46	38.15	36.10
Min-Max age	23.13–80.95	13.65–60.02	17.1–80.0
Ethnic distribution (%)			
Caucasian	94.2	66.1	56.2
North African	1.9	23.1	27.0
Central African	0.0	4.6	14.6
Asian	3.9	0.0	1.1
Miscellaneous	0.0	6.2	1.1

### Antigens and antigen-specific IFN-γ determinations

HBHA was purified from *Mycobacterium bovis* BCG as previously described [19], [20]. PPD (batch RT49) and recombinant ESAT-6 were obtained from the Statens Serum Institute, Copenhagen. PBMC were purified from fresh blood samples and *in vitro* stimulated as previously described [20], [21] with 2 µg/ml HBHA, 4 µg/ml PPD or 10 µg/ml ESAT-6. The IFN-γ concentrations were measured in 4-day cell culture supernatants by ELISA as previously described [20]. According to the manufacturer's instructions (www.cellestis.com), the QFT-G In-Tube (QFT-IT) assay was performed on whole blood, in contrast to the in-house IGRAs, and the cut-off value for a positive test was defined as IFN-γ≥0.35 IU/ml. For stimulation with the protein antigens (HBHA, ESAT-6, or PPD), we have chosen to incubate the PBMC for 96 h, rather than for 24 h, as recommended for the commercial QFT-IT, for two main reasons. Since proteic antigens were used for the ELISAs rather than peptides, as in QFT-IT, it is thought that they require some time for processing by antigen-presenting cells before they can stimulate T cells. Furthermore, prolonged incubation with antigen has recently been shown to increase the sensitivity of T cell tests for the detection of LTBI [23], probably because short *in vitro* incubation times in the presence of antigen preferentially result in the detection of effector T cells, whereas longer incubation times favor the detection of both effector and memory T cells [17], [18].

### Statistical analyses

Differences between the groups were assessed by the non-parametric Kruskal-Wallis test followed by the Dunn's multiple comparison test, or by the non-parametric Mann-Whitney U test. Receiver Operating Characteristic (ROC) curves were established for each mycobacterial antigen, and the areas under the curves (AUC) were compared to each other by using the following equation: z = |AUC_1_-AUC_2_|/√(Std Err_1_^2^+Std Err_2_^2^), with z>1.96 for a probability of 0.05 for making a type I error in rejecting the hypothesis that the 2 curves are similar. Correlation was analyzed by the non-parametric Spearman test and by a linear regression analysis. A value of *P*<0.05 was considered to be significant. All results were obtained with the Graphpad Prism Software version 4.0b.

## Results

### PPD-induced IFN-γ secretion

**Figure 1:**
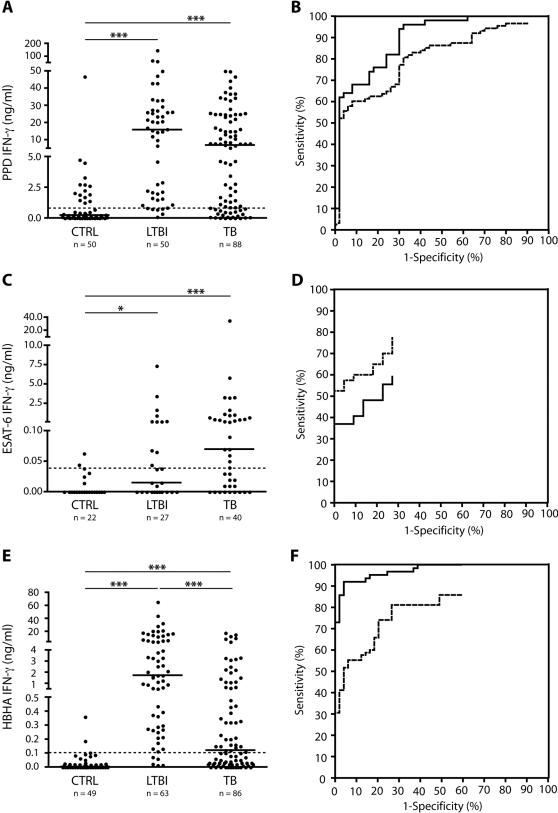
PPD, ESAT-6, and HBHA-induced IFN-γ concentrations and ROC analysis of the results. Results of the IFN-γ ELISA obtained for three different groups of subjects: non-infected controls (CTRL), subjects with latent *M. tuberculosis* infection (LTBI) and patients with active tuberculosis (TB) are represented on the left part of the figure. PBMC were *in vitro* stimulated with different antigens, PPD (A), ESAT-6 (C) and HBHA (E) and the IFN-γ concentrations were measured in 96 h cell culture supernatants. The horizontal dotted lines represent the cut-off for positive values as determined by ROC curves analysis, whereas the horizontal filled lines represent the medians of the results. ROC curves established for each antigen are represented on the right panels (PPD, B; ESAT-6, D; HBHA, F). The filled lines represent the curve established for LTBI compared to CTRL, whereas the dotted lines are those established for TB compared to CTRL. *, *P*<0.05; ***, *P*<0.001.

Since the LTBI subjects were defined on the basis of a positive TST reaction, we first evaluated the *in vitro* PPD-induced IFN-γ secretion by PBMC from LTBI subjects and TB patients compared to that from CTRL. As shown in Fig. 1A, LTBI subjects and TB patients secreted significantly more IFN-γ in response to PPD than CTRL (medians 15.83 ng/ml and 6.84 ng/ml, respectively, versus 0.23 ng/ml; *P*<0.001), whereas the median level of IFN-γ produced was not significantly different between the LTBI and TB groups. To evaluate the sensitivity and specificity of the *in vitro* PPD-IFN-γ test for the discrimination of LTBI from CTRL, a ROC curve was established and the AUC determined. As shown in Fig. 1B, the AUC was 0.89 (95% IC : 0.83–0.95), indicating both a good global accuracy of the PPD-induced IFN-γ production for discriminating LTBI from CTRL, and good agreement between a positive TST reaction and elevated PPD-specific IFN-γ secretion. A similar analysis comparing the TB patients with the CTRL gave an AUC of 0.80 (95% IC: 0.73–0.87). Using the ROC analysis, a cut-off IFN-γ concentration of 0.8 ng/ml was determined as being the most discriminatory between LTBI and TB versus the CTRL.

Based on this value, the PPD-induced-IFN-γ responses allowed us to differentiate LTBI from CTRL with a 90.0% sensitivity and a 70.0% specificity and to differentiate TB patients from the CTRL with a 75.0% sensitivity and a 70.0% specificity. The low specificity was a consequence of the positive responses from some CTRL, apparently mostly due to BCG vaccination (see below). Higher specificity of this test for LTBI may only be obtained at the expense of lower sensitivity.

### ESAT-6-induced IFN-γ secretion

Since ESAT-6 is largely restricted to members of the *M. tuberculosis* complex, measuring the IFN-γ responses to ESAT-6 should improve the specificity compared to the PPD-induced IFN-γ responses [24]. We therefore evaluated the ESAT-6-specific IFN-γ results in the three study groups, using the same methodology. The results shown in Fig. 1C confirmed that LTBI subjects and TB patients secrete higher amounts of IFN-γ in response to ESAT-6 than the CTRL (medians of 0.07 ng/ml for TB and below detectable levels for the two other groups; Fig. 1C). The ROC analysis of the LTBI and TB compared to the CTRL provided an AUC of only 0.70 (95% IC: 0.55–0.84) for LTBI and of 0.81 (95% IC: 0.70–0.91) for TB (Fig. 1D), compared to 0.89 and 0.80, respectively, obtained for the PPD-induced IFN-γ secretions. Comparisons by determining the z value of the curves obtained for ESAT-6 and PPD for LTBI indicated a lower global accuracy of ESAT-6 IGRA compared to PPD IGRA for the detection of LTBI (z = 2.41; *P*<0.05).

Using the ROC curves, a threshold of 0.040 ng/ml of IFN-γ was determined to best differentiate LTBI and TB from the CTRL group. Based on this value, the ESAT-6-induced-IFN-γ responses allowed us to differentiate LTBI from CTRL with a 40.74% sensitivity and a 90.91% specificity and to differentiate TB patients from the CTRL with a 60.00% sensitivity and a 90.91% specificity.

The last twenty LTBI subjects included in this study were also tested by using the QFT-IT test, as this test includes overlapping peptides of the *M. tuberculosis*-specific CFP-10 and TB 7.7 antigens in addition to ESAT-6 for lymphocyte stimulation (www.cellestis.com), which should increase the sensitivity of the blood test compared to stimulation by ESAT-6 alone. Ten out of the 20 subjects tested were positive with the QFT-IT test (IFN-γ≥0.35 UI), compared to 6 with the in-house ESAT-6 test, indicating that the QFT-IT test does appear to be more sensitive for the detection of LTBI than a test based on ESAT-6 alone. The sensitivity of the QFT-IT test remained nevertheless rather low for the detection of LTBI in this sampled population.

### HBHA-induced IFN-γ secretions

Given the low specificity of the PPD IFN-γ test and the low sensitivity of the ESAT-6 IFN-γ test for the detection of LTBI in this population, we assessed the diagnostic value of HBHA, a novel antigen, previously shown to induce significantly higher IFN-γ production by PBMC from LTBI compared to controls and TB patients [20], [21]. The previous findings were confirmed here (medians of 1.727 and 0.120 ng/ml for LTBI and TB, respectively; and below detectable median levels for the CTRL; *P*<0.001 for CTRL and LTBI; *P*<0.001 for CTRL and TB) (Fig. 1E). ROC analyses of LTBI and TB to CTRL gave an AUC of 0.97 (95% IC: 0.95–0.99) and 0.80 (95% IC: 0.72–0.87), respectively (Fig.1F). The AUC for the LTBI was significantly different from that obtained with PPD (z = 2.38 ; *P*<0.05), and from that obtained with ESAT-6 (z = 2.41 ; *P*<0.05), suggestive of a better global accuracy of HBHA-induced IFN-γ production compared to PPD- and ESAT-6-induced IFN-γ concentrations for the detection of LTBI. The cut-off value determined by the ROC analysis was 0.100 ng/ml, yielding a sensitivity of 92.06% and a specificity of 93.88% for the detection of LTBI. For TB, the sensitivity and specificity were 55.29% and 93.88%, respectively. However, due to the ELISA variation coefficient, 1.5% of the results obtained for LTBI and 4.5% of those obtained for TB may perhaps be considered as being indeterminate, as they fell between 0.080 and 0.120 ng/ml. Among the three antigens tested, HBHA provided thus the best overall diagnostic value for the detection of LTBI. Interestingly, among the 20 LTBI subjects that were also tested with the QFT-IT test, 17 scored positive for HBHA-induced IFN-γ, compared to only 10 positives for the QFT-IT test (see above).

**Figure 2:**
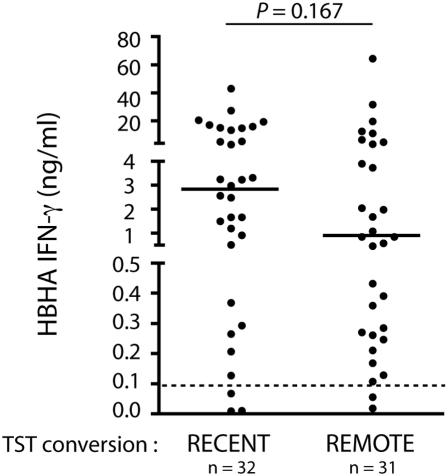
Comparison between the HBHA-induced IFN-γ concentrations in recently and in remote *M. tuberculosis* infected LTBI subjects. Blood samples were collected from LTBI subjects during the first two years (recent) or after two years (remote) of their TST conversion. Their PBMC were *in vitro* stimulated with HBHA, and the IFN-γ concentrations were measured in 96 h cell culture supernatants. No significant difference in the IFN-γ concentrations measured in the two groups was noted. The horizontal dotted line indicates the positivity cut-off of the test, and the horizontal filled lines indicate the medians.

The LTBI subjects were subdivided in two groups as defined in the Materials and Methods section. For the first group considered as being recently infected (n = 32), the blood sample was obtained within a maximum of 6 months after a TST conversion (21 household contacts and 11 health care workers). As a TST is performed once a year in health care workers, it is likely that the time elapsed between infection and blood sampling was at most 2 years. The second group of LTBI (n = 31) was considered to result from an infection in the more distant past, as their TST conversion had occurred more than 2 years before blood sampling (mean and SEM of the time between the TST and the blood sampling: 14.23 years, 9.68 years). No significant differences in the HBHA-induced IFN-γ concentrations were found between the two sub-groups of LTBI subjects (Fig. 2). The HBHA IGRA yielded sensitivities higher than 90% for the detection of both recently infected subjects and those with LTBI secondary to a remote *M. tuberculosis* infection (sensitivities of 90.62% and 93.54% for recent and remote infection, respectively). Among the 20 subjects tested with the QFT-IT test, most LTBI (n = 17) were the result of a remote infection. Among those 17 LTBI only 7 were positive in the QFT-IT test, whereas 15 were positive in the HBHA IGRA. In contrast, among the 3 recent TST converters, all three were positive in the QFT-IT test and two were positive in the HBHA IGRA. When these 17 LTBI subjects were followed by a longitudinal analysis, using 2 to 6 HBHA-based IGRAs spread over a total of 6 years, all were found positive at each time point (Table 1).

### Influence of BCG vaccination on the IFN-γ secretions

Although BCG vaccination is not routinely used in Belgium, approximately half of the CTRL in this study had nevertheless been vaccinated for various reasons more than 15 years before their inclusion in this study. This allowed us to evaluate the potential influence of BCG vaccination on the antigen-induced IFN-γ concentrations among the CTRL. PPD more frequently induced IFN-γ secretion by PBMC from BCG-vaccinated subjects (10 out of 22) than from non-vaccinated individuals (5 out of 26). This was not the case for the HBHA- (only 2 CTRL with an HBHA-induced IFN-γ secretion>0.1 ng/ml, one vaccinated and the other non-vaccinated) and for the ESAT-6-induced IFN-γ production (none in the CTRL).

A proportion of the LTBI subjects had also been previously vaccinated with BCG (n = 26). The comparison of the antigen-induced IFN-γ concentrations between the vaccinated LTBI and the non-vaccinated LTBI did not reveal significant differences for the IFN-γ responses induced by PPD-, ESAT-6 or HBHA.

### Pair-wise comparisons of the IFN-γ secretions induced by the different mycobacterial antigens

**Figure 3:**
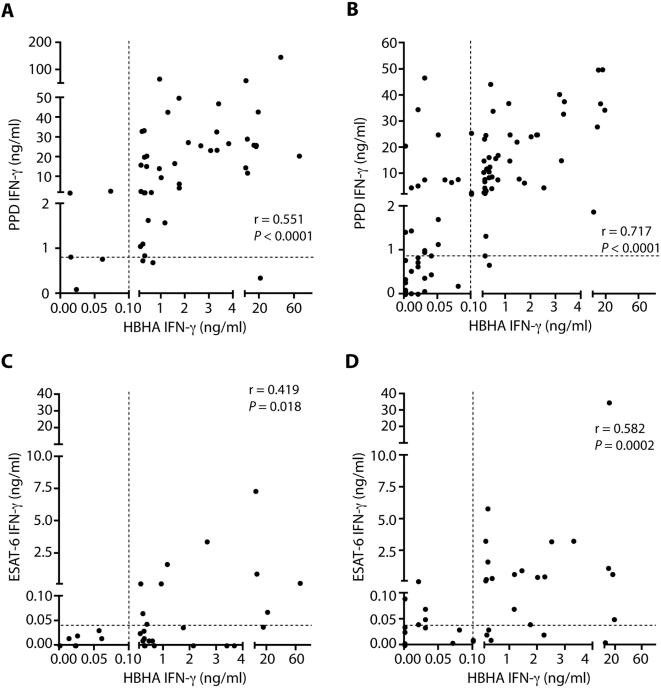
Correlations between the IFN-γ concentrations induced by different mycobacterial antigens. Results obtained for LTBI are represented on the left panels (A, C), and those obtained for TB are on the right panels (B, D). The degree of correlation between the *in vitro*-induced IFN-γ concentrations by the different mycobacterial antigens is represented for PPD and HBHA (A and B) and for ESAT-6 and HBHA (C and D). The dotted lines indicate the positivity cut-off for each test.

When the IFN-γ responses to PPD and ESAT-6 were compared pair-wise to the HBHA-specific responses, a moderate correlation was found for HBHA to PPD both in LTBI subjects (r = 0.55, *P*<0.0001) (Fig. 3A) and in TB patients (r = 0.71, *P*<0.0001) (Fig. 3B). A low or moderate correlation was found for HBHA to ESAT-6 in LTBI subjects (r = 0.41, *P* = 0.018) (Fig. 3C) and in TB patients (r = 0.58, *P* = 0.0002) (Fig. 3D).

Combining the values of the PPD- and the HBHA-induced IFN-γ secretions would not improve the diagnostic power for *M. tuberculosis* infection because of the low specificity of the PPD-specific response. Instead, we analyzed whether combining HBHA-induced and ESAT-6-induced IFN-γ secretions may help to increase sensitivity, while maintaining specificity at a high level. As shown in Fig. 3C, this combination did not increase the sensitivity for the LTBI subjects, as none of the LTBI subjects with a negative HBHA test was positive in the ESAT-6 test. In contrast, 45.16% of the LTBI subjects secreted IFN-γ in response to HBHA and not in response to ESAT-6 (Fig. 3C). However, combining HBHA-induced and ESAT-6-induced IFN-γ secretions allowed us to detect slightly more TB patients over the HBHA response alone, as 4/37 TB patients were only positive in the ESAT-6 test, and 5/37 were only positive in the HBHA test (Fig. 3D).

## Discussion

In this study, we examined the diagnostic value of HBHA, a novel mycobacterial antigen, for the detection of LTBI in a low-incidence country, where infection by NTM is rare and where BCG vaccination is not routinely used. We found that the *in vitro* HBHA-specific IGRA yielded a sensitivity of 92.06% and a specificity of 93.88% for the detection of LTBI, a substantially better sensitivity in this population than an ESAT-6-specific IGRA (40.74%, with a 90.91% specificity) and a substantially better specificity than a PPD-specific IFN-γ test (70%, with a similar sensitivity, 90.0%). In contrast, the ESAT-6 and HBHA responses were not significantly different for the diagnosis of acute TB. HBHA performed relatively poorly for the diagnosis of acute TB, as shown previously [21]. This is due at least partly to the induction of HBHA-specific regulatory T cells during active TB [25]. Combining the ESAT-6 results with the HBHA results did not improve sensitivity for LTBI. Low sensitivity of the ESAT-6 IGRA for the detection of LTBI, especially if carried out a long time after the point-source exposure has already been reported as being possibly related to the declining antigenic and bacterial load [26]. Similarly, although tested here only on 20 subjects, the QFT-IT test detected only 50% LTBI, compared to 85% detected in this series by the HBHA IGRA. The difference of performance between the two tests was especially pronounced when LTBI presumed to result from exposure in the remote past were diagnosed. In contrast, both tests were equally effective in recent TST converters.

An important consideration in this study is obviously the definition of the LTBI subjects enrolled. We have chosen rather stringent TST positive criteria, so that it is unlikely that the TST^+^ subjects were false positives because of NTM infection, although occasional mis-diagnosis of TST positivity due to NTM infection can obviously not formally be excluded. In addition, the study was carried out in Belgium, a country with a low TB incidence (10.9/100,000; www.fares.be). True LTBI resulting from remote exposure can only be studied in low- or intermediate-incidence areas, where re-exposure to *M. tuberculosis* is rare.

The influence of a previous BCG vaccination that might have given rise to false-positive TST results is also highly improbable, since 31 out of the 65 LTBI subjects were identified by the Occupational Medicine Department because of a TST conversion in the absence of a BCG vaccination at the time of the TST conversion. Furthermore, TST reactivity after BCG vaccination rarely lasts for more than 10 years in the absence of subsequent *M. tuberculosis* infection [27], making it also unlikely that the TST positivity of the remotely infected subjects was due to BCG vaccination in their childhood. In Belgium BCG vaccination is not routinely used, and only 7 of the subjects had been previously vaccinated, more than 15 years before their enrolment in the study. The contention that the HBHA response was not confounded by BCG vaccination is consistent with the fact that in the control group, no significant difference in the numbers of HBHA-responders was found between the vaccinated and the non-vaccinated subjects. In contrast, a higher number of positives were found in the vaccinated control group (10 out of 22) compared to the non-vaccinated group (5 out of 26) when the PPD responses were examined, probably accounting for the lower specificity of the PPD IGRA compared to the HBHA IGRA. The absence of influence of a previous BCG vaccination on the HBHA IGRA might be surprising, as HBHA is produced by all members of the *M. tuberculosis* complex, including *M. bovis* BCG [28]. Although the reasons for this difference between BCG vaccination and *M. tuberculosis* infection are not yet known, there are preliminary indications that the expression levels of the HBHA encoding gene are much lower in BCG than in *M. tuberculosis*, especially within cells in which the mycobacteria reside during latency (C. L., unpublished).

In this study, only the RT23 PPD was used for the TST, as this is the most widely used PPD in Europe. Previous studies have shown that the pattern of reactivity obtained with 2 TU of RT23 PPD is similar to that obtained with 5 TU of a different PPD, such as Tubersol [29]. Therefore, the findings reported here are expected to be similar if PPD preparations other than RT23 would have been used in the TST.

A major advantage of the HBHA IGRA reported here is the ability to detect LTBI resulting from a remote *M. tuberculosis* exposure. This may have practical consequences for TB control strategies. Although diagnosis and treatment of recently infected subjects deserves the utmost priority, because these subjects are at highest risk of developing active TB, detection of LTBI resulting from a remote infection may also be important. This is the case especially for health care workers [4] and for immunocompromised individuals, as immunosuppression because of either infection with HIV or of immunosuppressive treatment substantially increases the risk of TB reactivation [5]–[7]. Finally, in the long run, if eradication of TB is considered to be a realistic goal, especially in low-prevalence countries, LTBI subjects will have to be effectively diagnosed at a population-wide level [3], [6], [8].

A limitation of the present study is the relatively small sample size in the cross-sectional study design. However, a longitudinal follow-up performed for 17 LTBI subjects suggests that the subjects categorized as LTBI resulting from a remote infection remained positive and were thus true LTBI cases.

In conclusion, we provide here evidence that a HBHA-based IGRA may be useful for the detection of LTBI and is both substantially more sensitive for LTBI than an ESAT-6-based test, and substantially more specific than a PPD-based test. In addition, it is able to detect LTBI subjects with a remote infection that were missed by the QFT-IT test, a test proposed by the US Centers for Disease Control to replace the TST [30]. These LTBI subjects are important to detect in view of the increasing number of patients that require immunosuppressive therapy or that are infected with HIV and should therefore receive preventive anti-tuberculous treatment with high priority [6]. Thus, the current ESAT-6- and/or CFP-10-based IFN-γ IGRAs may underestimate LTBI, and our results indicate that the replacement of the TST by IGRAs should be considered with caution. The use of a HBHA IGRA may provide an attractive solution, by combining the operational advantages of an *in vitro* IFN-γ test with high sensitivity and specificity for LTBI.

However, one of the current challenges is that HBHA is a methylated antigen, and that the methylation is important for the HBHA-specific T-cell responses [21]. When recombinant, non-methylated HBHA is used instead of native, methylated HBHA, the sensitivity of the IGRA for the detection of LTBI decreases to 69.44% with a 88.46% specificity (data not shown). HBHA is thus not yet commercially available. However, a product development plan is currently being worked out, which should also allow us to test the diagnostic potential of this antigen as a skin test reagent. Furthermore, a prospective head-to-head comparison of the HBHA IGRA and the TST for the detection of LTBI among health care workers in Belgium will begin soon, as well as studies in immuno-suppressed populations, such as HIV-infected patients and patients starting infliximab therapy.
